# The Effect of COVID-19 on Neonatal Outcomes in a Community Hospital

**DOI:** 10.3390/jcm14020420

**Published:** 2025-01-10

**Authors:** Maria Martinez-Baladejo, Yemesrach Kerego, Allison R. Walker, Ashley Ohnona, Christina Scartelli, Clarke Stoltzfus, Ashley Graul, Dianne Jacobetz, Anna Ng-Pellegrino

**Affiliations:** 1 Department of Research and Innovation, St. Luke’s University Health Network, Bethlehem, PA 18015, USA; maria.martinez-baladejo@sluhn.org (M.M.-B.); allison.walker@sluhn.org (A.R.W.); anna.ngpellegrino@sluhn.org (A.N.-P.); 2 Obstetrics and Gynecology Department, St. Luke’s University Health Network, Bethlehem, PA 18015, USA; ashley.ohnona@sluhn.org (A.O.); ashley.graul@sluhn.org (A.G.); 3 Temple/St. Luke’s School of Medicine, St. Luke’s University Health Network, Bethlehem, PA 18015, USA; christina.scartelli@sluhn.org (C.S.);; 4 Department of Pediatrics, St. Luke’s University Health Network, Bethlehem, PA 18015, USA; dianne.jacobetz@sluhn.org

**Keywords:** SARS-CoV-2 infection, pregnancy, hypertension, developmental milestones, preeclampsia, infant growth parameters, neonatal, COVID-19, parturient, Ages and Stages Questionnaire

## Abstract

**Background**: Despite considerable research on pregnancy outcomes affected by severe acute respiratory syndrome coronavirus 2 (SARS-CoV-2) infection, the consequences for infants exposed to the virus in utero remain unclear. **Methods**: A retrospective cohort study was conducted, encompassing 392 mother–infant pairs delivered between April 2020 and July 2021 at a community hospital network in northeastern Pennsylvania, USA. Of these, 198 mothers had a confirmed SARS-CoV-2 infection during pregnancy, while 194 did not. Infant outcomes were compared between the two groups. **Results**: Pregnant women with a SARS-CoV-2 infection during their gestation exhibited higher rates of obesity (*p* = 0.04) with higher body mass indices (BMI) (*p* = 0.01), were more likely to be of Hispanic ethnicity (*p* = 0.01), and had a history of chronic hypertension (*p* = 0.05), as well as higher occurrences of postpartum depression (*p* = 0.01), gestational diabetes (*p* = 0.05), cesarean section (CS) rates (*p*< 0.001), and preeclampsia (*p* = 0.04). Among the infants reviewed, there was no statistical significance difference in developmental milestones at 2, 4, 6, 9, and 12 months of age between infants of parturients who tested positive for coronavirus disease 2019 (COVID-19) and infants of parturients without a positive COVID-19 test result. There was no statistically significant difference between the two groups in developmental outcomes, as measured by Ages and Stages Questionnaire (ASQ) scores at 9 months (*p* = 0.76) and at 18 months (*p* = 0.89). **Conclusions**: This study corroborates the adverse impact of SARS-CoV-2 infection on pregnant women, characterized by increased maternal comorbidities and adverse birth outcomes. No significant disparities in neonatal developmental milestones or growth outcomes were observed at birth; first office visit, or at 1, 2, 4, 6, 9, and 12 months of age.

## 1. Introduction

Women with severe acute respiratory syndrome coronavirus 2 (SARS-CoV-2) infection exhibit an augmented susceptibility to adverse obstetric outcomes, including heightened risks of preeclampsia (PEC), eclampsia, preterm birth (PTB), and cesarean section (CS) [1,2,3,4]. The underlying processes behind these connections remain an area of ongoing investigation, especially concerning the association between SARS-CoV-2 infection and PEC. While the causal relationship between SARS-CoV-2 and PEC is unclear [5], PEC itself is an independent risk factor for more severe SARS-CoV-2 infection requiring admission to an intensive care unit, mechanical ventilation, and maternal death [4,6].

Although direct transplacental SARS-CoV-2 infection has been reported to be rare, its potential to impact pregnancy outcomes through maternal systemic inflammatory responses remains a significant area of concern. This concern is well-founded, given that multiple maternal viral infections, such as cytomegalovirus, Zika virus, and rubella, are known to cause adverse developmental outcomes, such as fetal growth restrictions and developmental delays. These effects occur through mechanisms such as inflammation and placental dysfunction caused by immune system response [7,8].

Infants of mothers with confirmed SARS-CoV-2 infection during pregnancy are more likely to be admitted to the neonatal intensive care unit (NICU) [4] and exhibit sequelae extending beyond their NICU stay. Some researchers have noted that, at 1 year of age, infants born to SARS-CoV-2 infection may have increased risks of neurodevelopmental conditions, including disorders of speech and language, motor function, academic skills, and pervasive development disorders [9]. However, recent studies examining the correlation between the timing of maternal SARS-CoV-2 infection and adverse infant developmental outcomes show inconsistent findings. While some studies indicate that contracting the virus in the first or second trimester is strongly associated with adverse neonatal outcomes, others report no substantial differences across the trimesters [9,10,11]. These outcomes may depend on several variables, such as the severity of infection, maternal comorbidities, environmental or socioeconomic factors, and other confounders. Given the disparity and recent emergence of SARS-CoV-2, the current body of research surrounding coronavirus disease 2019 (COVID-19) and pregnancy warrants further investigation and follow-up to better understand its effects on maternal and fetal outcomes.

By focusing on a cohort of mother–infant pairs within a single community hospital network, using the extensively tested and validated Ages and Stages Questionnaire (ASQ) developmental screening tool [12,13] and standardized growth parameters, this study aims to contribute valuable insights into the potential impact of SARS-CoV-2 infection on neonatal growth and neurodevelopmental outcomes, providing a comprehensive understanding of the repercussions during the critical period of pregnancy and the initial stages of infant development.

## 2. Materials and Methods

After obtaining IRB approval, we performed a retrospective chart review from April 2020 to July 2021 of parturient patients at our community hospital system in Pennsylvania, USA. Through a review of the electronic medical records of 392 pregnant women, 198 patients had a positive SARS-CoV-2 test during their first, second, or third trimester of pregnancy (exposed group), and 194 patients did not have a positive test for the virus in their medical record (control group). The severity of COVID-19 symptoms was classified based on the Centers for Disease Control and Prevention (CDC) guidelines, which use clinical criteria such as respiratory rate and oxygen saturation levels [14]. The primary outcome was the incidence of PEC and PTB. Preterm birth was defined as delivery prior to 37 weeks gestation. Secondary outcomes included mode of delivery and the incidences of gestational diabetes mellitus (GDM), gestational hypertension (gHTN), preeclampsia (PEC), postpartum hemorrhage (PPH), polyhydramnios, pre-labor rupture of membranes (PROM), deep vein thrombosis (DVT), and post-partum depression. We limited our review of pre-existing conditions in the parturient population to chronic hypertension (cHTN), prior history of depression, thyroid disease, tobacco use during pregnancy, asthma, and pre-pregnancy diabetes.

Neonatal outcomes included newborn screening results (APGAR scores); need for initial resuscitation after birth (tactile stimulation, bulb suction, oxygen, or positive pressure ventilation [PPV]); developmental milestones at 9 and 18 months of age, as assessed by the ASQ-9 and ASQ-18 surveys (administered by the infants’ pediatricians during routine follow-up); and growth parameters during the first year of life (length, weight, and head circumference [HC]) and body mass index (BMI) based on data from birth, initial office visit (3–5 days after discharge from hospital), and 1, 2, 4, 6, 9, and 12 month well visits. The study team also reviewed the incidence of NICU admissions and referrals to developmental pediatrics. Data on developmental services were gathered through referrals documented in the electronic medical records.

We conducted separate unadjusted analyses to compare the demographic and clinical outcomes of mothers with confirmed COVID-19 versus those without COVID-19. We used SPSS version 29 (Armonk, NY, USA: IBM Corp) to analyze our data with independent samples t-tests for normally distributed continuous variables, Mann–Whitney rank sum tests for skewed continuous variables, and chi-square or Fisher’s exact tests, as appropriate, for categorical variables. For all comparisons, *p* < 0.05 denotes statistical significance, with no adjustment for multiple testing. Due to missing neonatal data and uneven subgroup samples, we conducted separate unadjusted analyses to compare outcomes between mothers and infants with and without COVID-19, using Student’s t-tests for normally distributed continuous variables, Mann–Whitney rank sum tests for skewed continuous variables, and chi-square or Fisher’s exact tests, as appropriate, for categorical variables.

Furthermore, a multifactorial analysis of variance (ANOVA) was performed to assess whether or not maternal SARS-CoV-2 infection was independently associated with ASQ-9 results (which was acceptably normally distributed), after controlling for PEC, BMI, race (White versus non-White), ethnicity (Hispanic versus non-Hispanic), advanced maternal age (AMA), and PTB. Although we planned to conduct multivariable logistic regression to assess independent contributors to PEC, we were unable to do so given the low event rate (*n* = 41) and subsequent insufficient subgroup samples; therefore, we conducted separate Student’s t-tests or chi-square tests, as appropriate, comparing cHTN, gHTN, GDM, tobacco use during pregnancy, BMI, race, ethnicity, AMA, and PTB.

## 3. Results

### 3.1. Parturient Outcomes 

Our study team compared 198 mothers who had a confirmed COVID-19 diagnosis within their first, second, or third trimester of pregnancy to 194 who did not have a positive COVID-19 test recorded in their medical record. Parturients infected with SARS-CoV-2 during their pregnancy were more likely to be obese (*p* = 0.04) with higher BMI (*p* = 0.01) and of Hispanic ethnicity (*p* = 0.01) (Table 1). There was a statistically significant association found in patients with a history of chronic hypertension between mothers with a positive SARS-CoV-2 infection compared to the control group (*p* = 0.05, Table 2). Parturients with confirmed SARS-CoV-2 infection also exhibited a strong association in the diagnoses of GDM (*p* = 0.05) and PEC (*p* = 0.04) (Table 3). In addition, there was a higher incidence of CS rates among parturients who tested positive for COVID-19 (*p*< 0.001) (Table 3). Lastly, based on separate unadjusted comparisons, the following variables were significantly associated with PEC: prior hypertension (*p* < 0.001); gestational hypertension (*p* = 0.005); advanced maternal age (AMA) (*p* = 0.024); and PTB (*p* = 0.016). 

We analyzed the distribution of COVID-19 symptom severity (mild or moderate/severe) across different trimesters of pregnancy. As per the CDC, mild symptoms refer to coughing, fatigue, fever/chills, headache, nausea, loss of sense of taste and smell, and minimal symptoms of respiratory distress. Moderate symptoms include shortness of breath and mild pneumonia diagnosis. Severe symptoms of COVID-19 are severe pneumonia, high oxygen requirements in need of hospitalization, organ failure, and death [14]. Among the 198 parturients who tested positive for SARS-CoV-2, the majority, 48.99%, acquired the infection during the second trimester, followed by 36.36% in the third trimester and 14.65% in the first trimester. Symptom severity did not significantly differ by the trimester of acquisition (*p* = 0.97) (Table 4). The distribution of symptom severity was noted to have a fairly consistent trend across trimesters, with mild symptoms accounting for 72.4% in the first trimester, 75.3% in the second trimester, and 73.6% in the third trimester.

### 3.2. Infant Outcomes 

The growth and development of 391 infants were reviewed in this study: 198 infants to parturients with confirmed SARS-CoV-2 infection and 193 infants to parturients without SARS-CoV-2 infection during gestation. One infant in the control group was excluded secondary to its unexpected death.

There were no statistical differences in APGAR scores and need for initial resuscitation after birth (tactile stimulation, bulb suction, oxygen, or positive pressure ventilation [PPV]) between infants of parturient mothers infected with SARS-CoV-2 and infants categorized in the control group (Table 5).

While evaluating growth parameters among infants in both groups at birth; the initial office visit; and 1, 2, 4, 6, 9, and 12 months well visits, statistical analysis was performed based on data that could be obtained via chart review for each growth parameter (length, weight, head circumference, and BMI) at that office visit (Table 6). After an extensive chart review of the infants in both groups, it was discovered that many infants in our study and control cohorts were born to mothers living in nearby large metropolitan cities, such as New York City and Philadelphia. Due to concerns about the initial COVID-19 pandemic wave, many parturients traveled away from their primary residences to give birth, seeking to avoid exposure to the virus. This subgroup of patients gave birth at our hospital system and subsequently returned to their primary residence over time. Hence, this posed a challenge for our study team in reviewing follow-up visits for infant development and growth. The n value in Table 6 for each cohort reflects the exact number of infants with data for each growth parameter.

After formal statistical analysis with the appropriate N, no differences in neonatal growth parameters of length, weight, and BMI during birth and follow-up office visits at the initial office visit and at 4, 6, 9, and 12 months were shown. A subgroup analysis found no significant association between the timing of maternal SARS-CoV-2 infection during pregnancy and neonatal/infant growth parameters (*p* > 0.05). Similarly, there was no significant correlation between the severity of maternal COVID-19 symptoms and these outcomes.

When reviewing ASQ questionnaires via chart review, 67% of infants to parturients in both study and control groups had ASQ 9 data, and 67% of infants of SARS-CoV-2 infected parturients and 66% of infants of non-SARS-CoV-2 infected parturients had ASQ 18 data to examine (Table 7). From both ASQ 9 and 18 survey data, there was no significant association with in utero COVID-19 exposure and infant developmental milestones based on the questionnaire (*p* = 0.76 for ASQ 9 answers and *p* = 0.89 for ASQ 18 answers).

Regarding referrals to pediatric developmental resources (such as physical therapy and speech therapy), data were available for 136 infants with suspected COVID-19 exposure from mothers and 126 infants with no suspected COVID-19 exposure from mothers. Of the 136 infants of SARS-CoV-2-infected mothers, 17.64% were referred to developmental pediatrics or early intervention with speech and physical therapy due to failure to achieve developmental milestones, compared to 15.9% of infants of women not infected with SARS-CoV-2. No statistical significance was noted between the study and control groups for SARS-CoV-2 in utero exposure and referrals to pediatric resources for development (Table 8).

We performed a multifactorial ANOVA test to evaluate the impact of pregnancy outcomes on ASQ 9 results. Although the following variables trended towards significance, they did not meet a *p*-value of statistical significance: PEC and white race (*p* = 0.76); SARS-CoV-2 infection, PEC, and AMA (*p* = 0.57); and SARS-CoV-2 infection, PEC, AMA, Hispanic ethnicity (*p* = 0.08).

## 4. Discussion

Consistent with prior studies [1,2,3,4,5,6,15], we observed that parturients who had a positive SARS-CoV-2 infection during their first, second, or third trimester of pregnancy had a higher association with an obesity diagnosis, prior history of chronic hypertension, and being of Hispanic ethnicity. In addition, pregnant parturients infected with SARS-CoV-2 exhibited higher rates of GDM, PEC, and CS. Lastly, consistent with known obstetrics risks, our PEC population was more likely to have cHTN [1].

We sought to investigate the potential association between in utero exposure to SARS-CoV-2 infection and delay in growth and developmental milestones in infants up to 1 year of age. In the limited number of patients at the fourth-, sixth-, ninth-, and twelve-month office well visits, likely due to logistical challenges preventing some infants from attending follow up visits after birth, we did not observe a significant difference in the growth parameters between the infant control group and study groups. Although not significant, our multivariate analysis suggests an association between ASQ-9 survey results in infants exposed to SARS-CoV-2 infection, advanced maternal age, and incidence of pre-eclampsia. There was also a suspected association between the ASQ-9 survey results of SARS-CoV-2 exposed infants and PEC, Hispanic ethnicity, and AMA. This finding suggests that it may not be PEC or SARS-CoV-2 infection alone that is responsible for these results, but rather the synergistic effect of these conditions existing comorbidly.

Although the mechanism by which in utero exposure to SARS-CoV-2 infection impacts neurodevelopmental outcomes is still being studied, several pathways have been proposed, including inflammatory responses triggering cytokine release with a potential to cross the placental barrier, hypoxia related to severe maternal SARS-CoV-2 infection, maternal stress hormones, and nutritional deficiencies [8,9]. For instance, a positive SARS-CoV-2 infection in a parturient may impact neurodevelopment, particularly in those with lower maternal choline levels during pregnancy [16]. Despite these concerns, our results align with recent studies, such as the ASPIRE trial, which reported no significant adverse neurodevelopmental outcomes in infants linked to in utero COVID-19 exposure [17].

SARS-CoV-2 infection during pregnancy has been linked to neurodevelopmental disorders, including autism spectrum disorder, cerebral palsy, and neuropsychiatric diseases (schizophrenia, bipolar disorder). Early environmental insult or stress can profoundly influence fetal organogenesis of the brain and neuronal axis [18]. Unfortunately, due to the relative infancy of the COVID-19 pandemic, elucidating the long-term consequence of maternal infection on their offspring will take years to decades. However, we can comment on the potential acute connection of maternal complication, fetal exposure to SARS-CoV-2 infection, and its effects. 

Inconsistencies in the literature further complicate the understanding of these associations. Eldow et al. conducted a retrospective cohort study involving 7772 infants and found that those exposed to SARS-CoV-2 infection in utero exhibited a greater likelihood of experiencing delayed developmental milestones, particularly among those exposed during the third trimester [9]. Similarly, a cohort study in China, encompassing 57 infants with prenatal SARS-CoV-2 exposure, revealed deficits in the social–emotional domain of neurodevelopmental testing at 3 months of age [19]. A prospective cohort study involving 298 infants exposed to SARS-CoV-2 in utero reported that approximately 10% exhibited developmental delay [10]. Interestingly, the risk of developmental delays was seemingly higher among infants exposed during the first and second trimesters, differing from Eldow et al.’s study. Furthermore, Wang et al.’s longitudinal study showed positive correlational findings, but it did not control for crucial confounding variables, such as instances of separation between mother and infant, and it lacked a control cohort, limiting the conclusiveness of the findings [10,20]. In contrast, Senem et al. [21] found no correlation between antenatal SARS-CoV-2 infection and delayed developmental milestones.

Various screening tools have been utilized in multiple studies to gauge developmental outcomes, such as the General Movement Assessment (GMA), Motor Optimality Score Revised (MOS-R), and the Ages and Stages Questionnaire (ASQ). Shah et al. administered the ASQ to 16–18-month-old infants of mothers who had a SARS-CoV-2 infection while pregnant, revealing an increased likelihood of developmental delays in infants, encompassing domains such as communication, fine and gross motor skills, problem-solving, or personal-social skills [22]. Similarly, Fajardo Martinez et al. [23] showed that infants exposed to COVID-19 in utero were more likely to have motor delays based on GMA and MOS-R evaluations at 6–8 months.

Conversely, limited research has explored the growth parameters of infants exposed to SARS-CoV-2 infection during fetal development. A retrospective chart review conducted by Shook et al. at the onset of the pandemic, involving 29,510 cases, revealed that maternal SARS-CoV-2 infections heightened the likelihood of infants having a higher body mass index (BMI) and subsequently receiving a cardiometabolic diagnosis [24]. Ockene et al. conducted a prospective study involving 149 infants, revealing that those exposed to the virus had lower birth weight initially but subsequently experienced accelerated weight gain during the first year of life [25].

Despite these concerning reports in the literature, it is imperative to consider various variables that may have influenced the outcome of the above research, including the timing of the studies and prematurity [26]. Fascinating insights were uncovered from a study of 272 mothers of infants born during the pandemic, irrespective of their exposure to SARS-CoV-2 in utero. Responses gathered through questionnaires administered at the six-month juncture indicated that the discerned impairments in neurodevelopmental trajectories across both exposed and unexposed groups could be ascribed to the unique conditions during gestation and in early childhood parenting occurring during the pandemic period [27]. This finding implies that the observed effects might be more closely linked to the overall context of the pandemic pregnancy experience rather than being directly attributable to the influence of SARS-CoV-2 exposure itself, underscoring that these infants were not solely impacted by in utero exposure to SARS-CoV-2, but also endured the effects of the pandemic-induced social isolation. For example, outside of the COVID-19 pandemic, instances of early separation between mother and baby have been associated with adverse consequences on infant brain development, parental psychosocial welfare, and the dynamic between parent and infant [28,29,30].

Furthermore, the synergistic impact of stress and social isolation experienced by mothers throughout the antenatal and postnatal periods could potentially elicit a harmful inflammatory response in the physiological systems of both maternal and infant entities. This, in turn, may have repercussions, not only on the immediate mental well-being of both parties but also on their enduring psychological health. Lastly, this interconnection may carry lasting developmental ramifications for the infants involved [31]. An additional aspect to consider is the potential presence of ascertainment bias, wherein mothers who have been exposed to SARS-CoV-2 may exhibit a heightened awareness of its enduring ramifications, leading them to proactively pursue medical assessment and guidance. 

### 4.1. Limitations

It should be noted that the sample sizes for both groups are relatively small, as our study team only focused on laboring patients in a single community hospital network during the COVID-19 pandemic between April 2020 and July 2021. Thus, our study outcomes may not be applicable or generalizable to a broader population. Additionally, our study relied on symptomatic testing protocols during the early pandemic, which may not have eliminated the possibility of misclassifying asymptomatic cases to the control group. Similarly, the lack of neonatal testing for SARS-CoV-2 infection and potential false-negative maternal tests could have led to the incorrect categorization of some patients.

Our study team focused on the growth of infants within their first year of life, by looking at length, height, weight, head circumference, and BMI. We also looked at stages of development using the ASQ-9 questionnaire at 9 and 18 months of age. Unfortunately, we could not reach any formidable conclusions or insight regarding infant development from our study. Family history of developmental disorders and risk factors for anoxic injury (abnormal fetal heart rate monitoring, arterial pH at the time of birth, and delivery indication were not considered. Social isolation, maternal infections, infant feeding choices, and separation between mothers and newborns were also not considered. 

Finally, we had a portion of the infants lost to follow-up from their ASQ surveys at 9 and 18 months, and our sample size was not large enough to identify rare outcomes, such as developmental delays related to SARS-CoV-2 infection. These limitations cannot be ignored and must be thoroughly considered when interpreting the results of this study. 

### 4.2. Future Research

We attribute the challenge of establishing a definitive link between fetal exposure to SARS-CoV-2 and neurodevelopmental delays or disorders to temporal factors and the compounding effects of social isolation, underscoring the necessity for further research. 

To address these gaps, future research should adopt a multidisciplinary clinical and translational approach, prioritizing the thorough examination of potential confounding variables. Prospective studies integrating comprehensive assessments of prenatal and postnatal growth parameters would enhance the depth of understanding in subsequent investigations. Additionally, due to the multifaceted impact of social isolation on maternal health, research elucidating the intricate interplay between maternal social isolation and developmental delays in infants is essential for a comprehensive understanding of this relationship. 

## 5. Conclusions

Extensive epidemiological studies have linked maternal infections and fetal exposure to the development of neurodevelopmental conditions. Despite this knowledge, the impact of a positive SARS-CoV-2 infection on parturients to infant growth and development remains uncertain. Our analysis did not reveal any causal relationship between SARS-CoV-2 infection in parturients and the growth or development of their infants. Although our findings align with recent studies reporting no significant adverse developmental outcomes related to in utero exposure to SARS-CoV-2 infection, conflicting reports in the literature may imply differences in study design and the tools used to screen neurodevelopment, highlighting the need for large-scale, multi-centric studies. Furthermore, incomplete ASQ data for both exposed and unexposed groups restricted our ability to draw definitive conclusions on the impact of SARS-CoV-2 infection on infants’ neurodevelopment. The findings for mothers mirrored the existing literature, indicating a need for continued exploration with larger sample sizes.

## Figures and Tables

**Table 1:** Maternal Demographics.

	Mothers with Positive COVID-19 Test (*n* = 198)	Mothers Without Positive COVID-19 Test (*n* = 194)	*p*-Value
Age (mean ± standard deviation)	29.3 ± 5.5	28.8 ± 5.7	0.39
AMA (>35 years), (*n*, %)	36 (18%)	30 (15.5%)	0.50
BMI (mean ± standard deviation)	33.9 ± 7.1	32.05 ± 6.6	**0.009**
Obesity (BMI > 30) (*n*, %)	136 (69%)	113 (58.9%)	**0.04**
White Race (*n*, %)	141 (70.1%)	144 (74.2%)	0.52
Hispanic Ethnicity (*n*, %)	72 (35.8%)	44 (22.7%)	**0.01**
Language (*n*, %)			
English	179 (89%)	180 (92.8%)	N/A
Spanish	18 (9%)	10 (5.2%)	
Marital Status (*n*, %)			
Married	97 (48.3%)	111 (57.2%)	N/A
Divorced	3 (1.5%)	0	
Single	98 (48.8%)	82 (42.3%)	
Significant Other	2 (1%)	0	

AMA, advanced maternal age; BMI, body mass index; N/A, not assessed. Bolded numbers indicate statistically significant results (*p* < 0.05).

**Table 2:** Pre-Existing Comorbidities of Parturients.

	Mothers with Positive COVID-19 Test (*n* = 198)	Mothers Without Positive COVID-19 Test (*n* = 194)	*p*-Value
Depression (*n*, %)	9 (12.4%)	17 (9.3%)	0.31
Anxiety (*n*, %)	27 (13.4%)	34 (17.5%)	0.26
Hypothyroidism (*n*, %)	18 (9%)	15 (7.7%)	0.66
Hyperthyroidism (*n*, %)	9 (4.5%)	10 (5.2%)	0.75
Tobacco Use During Pregnancy (*n*, %)	6 (3%)	7 (3.6%)	0.74
Asthma (*n*, %)	21 (10.4%)	25 (12.9%)	0.45
cHTN (*n*, %)	19 (9%)	8 (4.1%)	**0.05**
Diabetes (*n*, %)	4 (2%)	5 (2.6%)	0.70

cHTN, chronic hypertension. Bolded numbers indicate statistically significant results (*p* < 0.05).

**Table 3:** Pregnancy-Related Outcomes Stratified by SARS-CoV-2 Infection.

	With Positive COVID-19 Test (*n* = 198)	Without Positive COVID-19 Test (*n* = 194)	*p*-Value
Postpartum Depression (*n*, %)	8 (4%)	21 (10.8%)	**0.009**
gTHN (*n*, %)	20 (10%)	18 (9.3%)	0.82
GDM (*n*, %)	32 (15.9%)	18 (9.3%)	**0.05**
PEC (*n*, %)	27 (13.5%)	14 (7.2%)	
With severe features	13 (61.9%)	9 (64.3%)	**0.041**
Without severe features	8 (38.1%)	5 (35.7%)	
Mode of Delivery (*n*, %)			
CS	75 (37.3%)	33 (17%)	**<0.001**
NSVD	122 (60.7%)	154 (79.4%)	
VBAC	1 (0.5%)	0	
Anesthesia (*n*, %)			
Epidural	121 (60.2%)	164 (84.5%)	**<0.001**
Spinal	57(28.4%)	28 (14.4%)	
General Anesthesia	1 (0.5%)	0	
No Anesthesia	15 (7.5%)	2 (1%)	
PPH (*n*, %)	3 (1.5%)	2 (1%)	0.68
DVT (*n*, %)	0	1 (0.5%)	0.49
Polyhydramnios (*n*, %)	8 (4%)	6 (3.1%)	0.63
PTB	17 (8.4%)	7 (3.6%)	0.13
PPROM (*n*, %)	3 (1.5%)	5 (2.6%)	0.44
Length of Stay, Days (median, range)	2 (1–8)	2 (1–8)	1.00
Gestational Age at Delivery (median, range)	39 (29.4–42.2)	39.2 (29.2–41.1)	1.00

gTHN, gestational hypertension; GDM, gestational diabetes mellitus; PEC, pre-eclampsia; CS, cesarean section; NSVD, normal spontaneous vaginal delivery; VBAC, vaginal birth after cesarean section; PPH, postpartum hemorrhage; DVT, deep venous thrombosis; PTB, preterm birth; PPROM, preterm premature rupture of membrane. Bolded numbers indicate statistically significant results (*p* < 0.05).

**Table 4:** COVID-19 Positive Parturients and Severity of COVID-19 symptoms based on Trimester.

	Overall	No Symptoms	Mild Symptoms	Moderate to Severe Symptoms	*p*-Value
Trimester of SARS-CoV-2 infection acquisition					0.970
First	29 (14.65%)	2 (22.22%)	21 (14.29%)	6 (14.29%)	
Second	97 (48.99%)	4 (44.44%)	73 (49.66%)	20 (47.62%)	
Third	72 (36.36%)	3 (33.33%)	53 (36.05%)	16 (38.10%)	

**Table 5:** Infant-Related Outcomes Stratified by SARS-CoV-2 Infection.

	Infants with COVID-19 Exposure (*n* = 198)	Infants without COVID-19 Exposure (*n* = 193)	*p*-Value
NICU admissions (*n*, %)	18 (9%)	9 (4.7%)	0.09
1 min APGAR (median, range)	8 (1–9)	9 (3–9)	0.11
5 min APGAR (median, range)	9 (4–9)	9 (7–10)	0.07
Tactile Stimulation (*n*, %)	184 (91.1%)	184 (94.8%)	0.21
Bulb Suction (*n*, %)	189 (93.6%)	185 (95.4%)	0.71
Oxygen (*n*, %)	11 (5.4%)	8 (4.1%)	0.55
PPV (*n*, %)	5 (2.5%)	4 (2.1%)	0.79
Placental Complications (*n*, %)	76 (37.6%)	71 (36.6%)	0.52

NICU, neonatal intensive care unit; PPV, positive pressure ventilation.

**Table 6:** Infant Growth Outcomes.

	Infants with COVID-19 Exposure (*n* = 198)		Infants Without COVID-19 Exposure (*n* = 193)		*p*-Value
	*n*	Median (Range)	*n*	Median (Range)	
Birth Length (cm)	198	49.5 (43.18–55.88)	193	49.5 (40.64–55.88)	0.26
Birth Weight (g)	198	3277 (1679–4593)	193	3310 (1704–4400)	0.33
Birth HC (cm)	185	34 (29–49.5)	181	33.5 (28–37.5)	0.24
First office visit					
Length (cm)	141	49.2 (43.18–116.08)	119	49.5 (43.2–56.5)	1.61
Weight (g)	154	3161 (2115–4300)	139	3175 (2098–4366)	0.64
HC (cm)	129	34 (13.5–45.7)	108	34.3 (28.6–37.5)	0.58
BMI (kg/m²)	150	12.91 (10.18–16.59)	139	13.01 (9.84–16.11)	0.92
One-Month Visit					
Length (cm)	139	53.49 (46.99–56.69)	123	54 (47–59.7)	0.79
Weight (g)	142	4252 (2807–5613)	126	4335 (2920–6124)	0.35
HC (cm)	138	37 (14–41)	122	37.5 (31.8–40.6)	**0.01**
BMI (kg/m^2^)	141	14.86 (9.63–19.35)	123	14.76 (10.77–18.55)	0.63
Two-Month Visit					
Length (cm)	144	57.15 (51.41–150.11)	132	57.85 (52.1–65.4)	0.18
Weight (g)	146	5307.5 (3710–7680)	132	5450 (3737–6771)	0.09
HC (cm)	144	39 (29.9–42.4)	130	39.4 (34.3–42.5)	**0.04**
BMI (kg/m^2^)	144	16.09 (12.44–20.45)	132	16.36 (11.89–20.6)	0.61
Four-Month Visit					
Length (cm)	138	63.5 (54.991–71.12)	123	63.5 (41.7–69.9)	0.18
Weight (g)	138	6792.5 (4875–9259)	124	6923.5 (5041–9696)	0.16
HC (cm)	136	41.9 (15–66)	122	41.9 (16.5–45)	0.27
BMI (kg/m^2^)	138	16.8 (12.85–24.91)	123	17.06 (13.29–21.44)	0.28
Six-Month Visit					
Length (cm)	135	67.31 (61.11–98.43)	118	67.4 (61.6–73.7)	0.70
Weight (g)	135	7830 (1060–10,900)	119	8023 (5931–11,100)	0.50
HC (cm)	133	43.6 (18–47)	116	43.65 (38.1–114.3)	0.78
BMI (kg/m^2^)	134	17.23 (13.75–22.9)	118	17.36 (14.14–22.38)	0.89
Nine-Month Visit					
Length (cm)	124	72.0598 (61.09–78.74)	111	72.4 (65.9–78.7)	0.33
Weight (g)	126	9072 (6396–12,100)	111	9202 (6401–13,400)	0.28
HC (cm)	123	45.5 (17.5–49.5)	109	46 (40.6–113)	0.10
BMI (kg/m^2^)	124	17.43 (13.75–23.66)	111	17.67 (14.35–23.14)	0.51
Twelve-Month Visit					
Length (cm)	125	75.99 (68.30–83.82)	119	76 (55.9–83.2)	0.25
Weight (g)	125	9724 (7008–12,556)	118	10,000 (7757–13,400)	0.15
HC (cm)	120	46.55 (19–49.5)	116	46.6 (40.6–49.5)	0.22
BMI (kg/m^2^)	124	17.06 (13.06–25.46)	118	17.33 (14.3–21.51)	0.39

HC, head circumference; BMI, body mass index; Based on separate Student’s *t*-tests, Mann Whitney rank sums tests, or chi-square or Fisher’s exact tests, as appropriate, with missing data points for certain outcomes. Bolded numbers indicate statistically significant results (*p* < 0.05).

**Table 7:** ASQ 9 and 18 Questionnaire Data in Infants.

Outcome	Infants Exposed to COVID-19 (*n* = 198)	Infants Without COVID-19 Exposure (*n* = 193)	*p*-Value	Odds Ratio
ASQ-9 results				
Total evaluated	134	130		
Passed	124 (91.5%)	119 (91.54%)	0.76	0.87
Failed	10 (8.46%)	11 (8.46%)		
Missing data	64	63		
ASQ-18 results				
Total evaluated	132	127		
Passed	111 (84.1%)	106 (83.5%)	0.89	1.05
Failed	23 (17.4)	21 (16.5%)		
Missing data	66	66		

**Table 8:** Comparison of Referrals to Developmental Pediatrics between COVID-19 Exposed and Unexposed Infants.

Referrals to Pediatric Developmental Resources	Infants Exposed to COVID-19 (*n* = 198)	Infants Without COVID-19 Exposure (*n* = 193)	*p*-Value	Odds Ratio
Total evaluated	136	126		
Referred	24 (17.64%)	20 (15.9%)	0.70	1.14
No referral	112 (82.35%)	106 (84.1%)		
Missing data	62	67		

## Data Availability

This research was collected from the electronic medical records at our single institution and, due to the linking of maternal/neonatal outcomes and identifying patient information, these data are not publicly available for future research collaborations or studies. Please direct any inquiries to the corresponding author.
